# Pathophysiological power of improper tonic GABAA conductances in mature and immature models

**DOI:** 10.3389/fncir.2013.00170

**Published:** 2013-10-24

**Authors:** Kiyoshi Egawa, Atsuo Fukuda

**Affiliations:** ^1^Department of Neurology, Massachusetts General HospitalCharlestown, MA, USA; ^2^Department of Pediatrics, Hokkaido University Graduate School of MedicineSapporo, Japan; ^3^Department of Neurophysiology, Hamamatsu University School of MedicineHamamatsu, Japan

**Keywords:** GABA, GAT, extrasynaptic, ambient, transporter, tonic inhibition, neurological disease, GABA_A_ receptor

## Abstract

High-affinity extrasynaptic gamma-aminobutyric acid A (GABA_A_) receptors are tonically activated by low and consistent levels of ambient GABA, mediating chronic inhibition against neuronal excitability (tonic inhibition) and the modulation of neural development. Synaptic (phasic) inhibition is spatially and temporally precise compared with tonic inhibition, which provides blunt yet strong integral inhibitory force by shunting electrical signaling. Although effects of acute modification of tonic inhibition are known, its pathophysiological significance remains unclear because homeostatic regulation of neuronal excitability can compensate for long-term deficit of extrasynaptic GABA_A_ receptor activation. Nevertheless, tonic inhibition is of great interest for its pathophysiological involvement in central nervous system (CNS) diseases and thus as a therapeutic target. Together with the development of experimental models for various pathological states, recent evidence demonstrates such pathological involvements of tonic inhibition in neuronal dysfunction. This review focuses on the recent progress of tonic activation of GABA_A_ conductance on the development and pathology of the CNS. Findings indicate that neuronal function in various brain regions are exacerbated with a gain or loss of function of tonic inhibition by GABA spillover. Disturbance of tonic GABA_A_ conductance mediated by non-synaptic ambient GABA may result in brain mal-development. Therefore, various pathological states (epilepsy, motor dysfunctions, psychiatric disorders, and neurodevelopmental disorders) may be partly attributable to abnormal tonic GABA_A_ conductances. Thus, the tone of tonic conductance and level of ambient GABA may be precisely tuned to maintain the regular function and development of the CNS. Therefore, receptor expression and factors for regulating the ambient GABA concentration are highlighted to gain a deeper understanding of pathology and therapeutic strategy for CNS diseases.

## INTRODUCTION

Neurotransmission comprises both excitatory and inhibitory signals. Therefore, understanding the mechanisms underlying the imbalance of these signals that are found in pathologies of the central nervous systems (CNSs) are vital in pursuing specific therapeutic strategies. In inhibitory neurotransmitter systems, gamma-aminobutyric acid (GABA) A receptor (GABA_A_)-mediated synaptic transmission is particularly important because its fast, ligand-gated inhibitory conductance allows fine homeostatic tuning of excitation-inhibition balance (see review Farrant and Nusser, 2005; Olsen and Sieghart, 2008).

Accumulating evidence has revealed that the function and distribution of the GABA_A_ receptor are remarkably diverse because of their subunit assembly. Some GABA_A_ receptor isoforms have been shown to be expressed outside synapses, and tonically activated by low concentrations of GABA (i.e., tonic inhibition) existing in the extrasynaptic space (Mody and Pearce, 2004; Farrant and Nusser, 2005). Although the proportion of GABA_A_ receptor subtypes mediating tonic inhibition is estimated to be of minor abundance as compared with that mediating synaptic inhibition (Mohler et al., 2002), this effect nevertheless provides strong inhibitory force by shunting the electrical signal transmission. With increased knowledge about its functional significance, an alteration in tonic inhibition has received great attention as a mechanism underlying the pathophysiology of various CNS disorders. Its distinct properties and pharmacology may thus provide new therapeutic strategies for overcoming limitations of conventional GABA_A_ receptor modulators. Thus far, deregulation of tonic inhibition has been shown to be a mechanism responsible for underlying a variety of CNS pathologies, including epilepsy, neurodevelopmental disorders, cognitive dysfunctions, and psychiatric disorders (see review, Brickley and Mody, 2012; Hines et al., 2012).

In addition to tonic inhibition induced by GABA generated by synaptic spillover, another form of tonic conductance occurs before synaptic formation. This tonic conductance is depolarizing because the intracellular chloride (Cl^-^) concentration is high in immature neurons due to the balance of Cl^-^ transporters (Owens et al., 1996; Yamada et al., 2004; for review, see Ben-Ari, 2002). The release of GABA in the milieu occurs via non-vesicular mechanisms (Demarque et al., 2002; Manent et al., 2005; for review, see Owens and Kriegstein, 2002). This tonic GABA_A_ receptor-mediated conductance is considered to be involved in a variety of developmental events, such as neurogenesis (LoTurco et al., 1995; Haydar et al., 2000; Andäng et al., 2008), migration (Behar et al., 1996,^1998^,^2000^,^2001^; López-Bendito et al., 2003; Cuzon et al., 2006; Heck et al., 2007; Bortone and Polleux, 2009; Denter et al., 2010; Inada et al., 2011; Inoue et al., 2012) and synaptogenesis (Nakanishi et al., 2007; Wang and Kriegstein, 2008). Based on these findings, perturbation of tonic depolarization could also result in brain mal-development.

In this review, we provide an overview on the advancement in knowledge about the role of dysregulation of tonic inhibition in the pathophysiology of various CNS diseases (with the exception of epilepsy, which will be reviewed independently in this issue), and its involvement in their therapeutic strategies. Furthermore, we review the latest findings of tonic depolarization underlying brain mal-development (with the exception of the hippocampus, which will be reviewed independently in this issue).

## RECEPTOR COMPOSITION AND DISTRIBUTION OF TONIC INHIBITION

Gamma-aminobutyric acid A receptors are assembled from a family of 19 homologous subunit gene products (six α subunits, three β subunits, three γ subunits, three ρ subunits, and one each of the ε, δ, θ, and π subunits) and form mostly hetero-oligomeric pentamers (for review, see Olsen and Sieghart, 2008). Most GABA_A_ receptor subtypes are formed from two copies of a single α, two copies of a single β, and one copy of another subunit (γ, δ, or ε). Each subunit combination has a distinct distribution pattern in terms of subcellular domains. The δ subunit, generally partnered with the α4 (forebrain predominant) and α6 (cerebellum predominant) subunits, are shown to be exclusively localized in extrasynaptic membranes in various neuronal cells, including cerebellar granule cells (CGCs), dentate gyrus granule cells, neocortical layer 2/3 pyramidal cells (Nusser et al., 1998; Nusser and Mody, 2002; Wei et al., 2003), interneurons in the neocortex and hippocampus (Semyanov et al., 2003; Krook-Magnuson et al., 2008), thalamic relay neurons (Cope et al., 2005), medium spiny neurons of the striatum (Ade et al., 2008), and dorsal horn spinal neurons (Bonin et al., 2011). The δ subunit-containing GABA_A_ (δ-GABA_A_) receptor shows high affinity and slow desensitization to GABA (Saxena and MacDonald, 1996), which allows tonic activation in response to low concentration of ambient GABA in extrasynaptic space (though see Bright et al., 2011).

The α5 subunit-containing GABA_A_ receptors, which presumably do not include the δ subunit, are also expressed in extrasynaptic membranes. The α5 subunit is predominantly detected in hippocampal pyramidal neurons (Sperk et al., 1997), showing higher GABA sensitivity compared with α1 GABA_A_ receptors, which allows tonic inhibition in hippocampal pyramidal neurons (Caraiscos et al., 2004; Glykys and Mody, 2006). The α1 and α5 subunit-mediated tonic inhibition is also shown in cortical principal neurons in layer 2/3 and layer 5, respectively (Yamada et al., 2007). In line with the predominant distribution of extrasynaptic α5 subunit-containing GABA_A_ receptors (Brunig et al., 2002; Crestani et al., 2002; Caraiscos et al., 2004), perisynaptic α5 subunits also mediate the GABA spillover component of slow phasic inhibitory currents (Prenosil et al., 2006). The contribution of such a slow inhibitory synapse transmission to the signal computation may be a future issue to explore with further studies.

The other assemblies (e.g., α1γ2 subunit-containing receptor), which mainly mediate synaptic inhibition, also exist in the extrasynaptic membrane (Kasugai et al., 2010) and can contribute to tonic inhibition because their specific agonist, zolpidem, amplifies the tonic currents (Semyanov et al., 2003; Yamada et al., 2007). High affinity αβ subunit-containing GABA_A_ receptors have also been shown to mediate tonic inhibition in rat hippocampal pyramidal neurons (Mortensen and Smart, 2006). Nevertheless, it is now clear that the GABA_A_ receptor subtypes having the δ or α5 subunit are the dominant forms which are responsible for mediating tonic inhibition in the mammalian brain, including humans (Scimemi et al., 2006).

## REGULATION OF TONIC INHIBITION

After the finding of tonic inhibition in the mature brain (Kaneda et al., 1995), its functional significance for regulating network excitability was subsequently revealed. In CGCs, blocking tonic inhibition decreases membrane shunting (Brickley et al., 1996) and increases information flow from granule cells to Purkinje cells (Hamann et al., 2002). In thalamic relay neurons or hippocampal interneurons, baseline membrane potentials and network oscillation are modulated (Cope et al., 2005; Song et al., 2011). Deregulation of tonic inhibition caused by a lack of synaptic plasticity-associated proteins has been shown in several mice models of neurological disorders (Curia et al., 2009; Olmos-Serrano et al., 2010; Egawa et al., 2012). However, the pathophysiological significance of long-term modification of extrasynaptic GABA_A_ receptor activation can be masked by compensatory mechanisms (Brickley et al., 2001; Wisden et al., 2002). Some adaptive regulation responses could be attributed to the modification of GABA_A_ receptor trafficking and/or clustering on the membrane surface (Saliba et al., 2012; see review Hines et al., 2012).

Progress in the development of pharmacological agents that predominantly modulate extrasynaptic GABA_A_ receptors (summarized in Table 1) has contributed to the uncovering of the pathological significance of dysregulated tonic inhibition. Consequently, large efforts have been made to use these agents for clinical use. The negative allosteric modulator of the α5 GABA_A_ receptor, α5IA, has recently been shown to improve ethanol-induced impaired performance in healthy subjects (Nutt et al., 2007). However, its use in a clinical trial was stopped due to renal toxicity (Atack, 2010). Another negative allosteric modulator of the α5 subunit, RO4938581, is presently under a phase 1 clinical trial for Down syndrome (the study is sponsored by Roche, and its outcomes have been made publicly available). The selective orthosteric agonist of the δ-GABA_A_ receptor, 4,5,6,7-tetrahydroisoxazolo(5,4-c)pyridin-3-ol (THIP, or also known as gaboxadol) was used as an hypnotic drug, reaching a phase 3 trial before its cessation because of side-effects, such as hallucination and disorientation (the study is sponsored by Merck, and its outcomes have been made publicly available). However, THIP has been recently shown to be effective against behavioral and motor dysfunction in mouse models of autism spectrum disorders (Olmos-Serrano et al., 2011; Egawa et al., 2012). Therefore, further cumulative evidence may hopefully renew an interest in using THIP for these diseases, which have no effective therapeutic strategies.

**Table 1:** Selective modulators for extrasynaptic GABA_**A**_ receptor.

δ-Subunit selective		
THIP (4,5,6,7-tetrahydroisoxazolo (5,4-c)pyridin-3-ol; gaboxadol)	Selective orthosteric agonist of δ-GABA_A_ receptor, a restricted analog of muscimol	Krogsgaard-Larsen et al. (2004)
AA29504	Positive allosteric modulator of δ-GABA_A_ receptor, analog of Kv7 channel opener, retigabine	Hoestgaard-Jensen et al. (2010), Vardya et al. (2012)
DS2	Positive allosteric modulator of δ-GABA_A_ receptor, poor blood–brain barrier permeability	Wafford et al. (2009), Jensen et al. (2013)
THDOC	Positive allosteric modulat,or of δ-GABA_A_ receptor, an endogenous neurosteroid	Bianchi and MacDonald (2003)
**α5-Subunit selective**		
RO4938581	Negative allosteric modulator of α5-GABA_A_ receptor, now entering to phase1 trial for Down syndrome	Ballard et al. (2009)
α5IA	Negative allosteric modulator of α5-GABA_A_ receptor, development has stopped due to renal toxicity	Atack (2010)
MRK-016	Negative allosteric modulator of α5-GABA_A_ receptor, more potent than α5IA	Atack et al. (2009)
L-655,708	Negative allosteric modulator of α5-GABA_A_ receptor	Atack et al. (2006)
SH-053-20F-R-CH3	Positive allosteric modulator of α5-GABA_A_ receptor	Gill et al. (2011)

Because extrasynaptic GABA_A_ receptors are activated by ambient GABA in the extrasynaptic space, regulation of ambient GABA concentrations must therefore be an important factor for determining the degree of tonic inhibition. The concentration of ambient GABA has been estimated to be very low, less than 300 nM in both* in vivo* (Kennedy et al., 2002) and *in vitro* (Wu et al., 2003; Santhakumar et al., 2006) conditions. A considerable part of ambient GABA may be derived from spillover from the synaptic cleft (Glykys and Mody, 2007). Thus, presynaptic functions, such as GABA synthesis and GABA release, should also regulate tonic GABA conductance. To date, reverse mode operation of GABA transporters (Wu et al., 2003; Heja et al., 2012) and GABA release via bestrophin1 anion channel (Lee et al., 2010) have been proposed as mechanisms underlying the non-vesicular release of GABA, both of which are currently under debate (Diaz et al., 2011; Kersante et al., 2013).

Regardless of the source of ambient GABA, GABA transporters play a pivotal role in regulating tonic inhibition. In the rat hippocampus, presynaptically located GABA transporter 1 (GAT1) and astrocytic GAT3 (corresponding to GAT4 in mice) synergistically modulate the ambient concentration of GABA via its uptake from the extrasynaptic space (Egawa et al., 2013; Kersante et al., 2013; Song et al., 2013). *In vivo* analysis has shown that neuronal GAT1 plays a predominant role under resting conditions and that the uptake of GABA by astrocytic GAT3 occurs during membrane depolarization (Kersante et al., 2013). In general, GAT1 and GAT3 is expressed in presynaptic neurons and astrocytes, respectively. However, this expression pattern is not consistent throughout the brain regions. For example, thalamic GAT1 and GAT3 are exclusively expressed in astrocytes, but not in presynaptic neurons (De Biasi et al., 1998), and thus the regulatory mechanisms for ambient GABA in the brain can differ depending on the region. Recently, compromised function of glial GATs (associated with increased tonic inhibition), has been shown to underlie the pathophysiology of absence epilepsy (Cope et al., 2009) and prevent recovery after stroke (Clarkson et al., 2010). Insufficient (Chiu et al., 2005) or excessive (Egawa et al., 2012) amounts of GAT1 can cause cerebellar dysfunction, at least in part, to deregulation of tonic inhibition.

**FIGURE 1:**
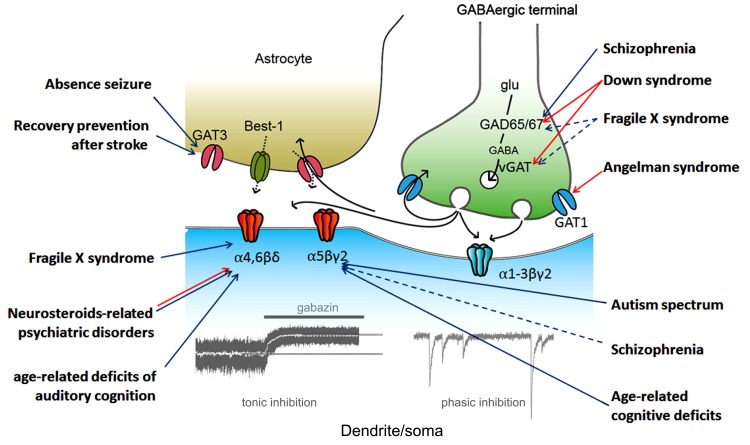
**Regulation of tonic inhibition and CNS disorders**. Aberrant tonic inhibition is suspected to be a mechanism underlying the neuronal dysfunctions in the listed disorders based on at least one of the following: (1) a direct measurement of tonic inhibition in model animals, (2) the use of extrasynaptic gamma-aminobutyric acid A (GABA_A_) receptor selective modulators in model animals, and (3) evaluation of α5 subunit expression in living human subjects (via positron emission tomography). Colored arrows indicate the proposed protein/subunit responsible for aberrant tonic inhibition (blue, down-regulation; red, up-regulation; black, GABA signaling during synaptic transmission). Dashed arrows indicate pathways still under debate. vGAT, vesicular GABA transporter; Best-1, bestrophin-1; glu, glutamate.

A Schematic drawing of the regulation of tonic inhibition with some implications for CNS disorders possibly associated with dysfunction of tonic inhibition (details will be shown below) is shown as **Figure 1**.

## SENSORY FUNCTIONS

Tonic GABA conductances exist within ascending sensory pathways on each level, including spiny neurons of the dorsal horn in the spinal cord (Bonin et al., 2011), thalamic relay neurons (Cope et al., 2005; Richardson et al., 2011), granule cells of the olfactory bulb (Labarrera et al., 2013), the brain stem auditory pathway (Campos et al., 2001), and the primary sensory cortex (Yamada et al., 2007; Krook-Magnuson et al., 2008; Imbrosci et al., 2012). Tonic inhibition is thought to be important for sensory processing because it reduces the impact of low frequency excitation, thereby increasing its signal-to-noise ratio.

4,5,6,7-Tetrahydroisoxazolo(5,4-c)pyridin-3-ol (THIP) acts as an anti-nociceptive in rodent models of acute pain (Enna and McCarson, 2006; Munro et al., 2008; Bonin et al., 2011). The anti-nociceptive effect is absent from δ- or α4-GABA_A_ receptor null mutant mice (Chandra et al., 2006; Bonin et al., 2011), indicating that tonic inhibition plays major roles in the regulation of acute nociception. The effects of THIP have been attributed to actions at supraspinal targets, such as thalamic relay neurons (Zorn and Enna, 1985; Rode et al., 2005; Chandra et al., 2006). Tonic GABA currents mediated by the δ subunit also exist in the spinal cord dorsal horn (Bonin et al., 2011). Studies on δ subunit knock-out (KO) mice indicate that tonic inhibition likely regulates the late phase of nociceptor activity, thus corresponding to central sensitization (Bonin et al., 2011). This response may result from amplification of tonic inhibition caused by activity-dependent GABA spillover or induction of neurosteroids during early phase of nociception. Nociceptor activity in early phase was shown to be comparable between wild type (WT) and δ-GABA_A_ receptor KO mice (Bonin et al., 2011). Therefore, a tonic inhibition in spinal cord neurons may also contribute to regulating central sensitization and acute nociception.

In the auditory thalamus, the medial geniculate body is essential for processing acoustic information. A recent study has demonstrated that age-related loss of inhibition in this area is more dominant in tonic rather than phasic inhibition (Richardson et al., 2013). Decreased expression of α4δ-GABA_A_ receptors and down-regulation of glutamic acid decarboxylase (GAD) 67 have been found to be possible mechanisms underlying this difference (Richardson et al., 2013). Because the efficacy of auditory coding is directly correlated with the strength of GABAergic inhibition (Gleich et al., 2003; de Villers-Sidani et al., 2010), enhancement of tonic inhibition was proposed to be a potential strategy against age-related decline of auditory cognition. A reduction of tonic inhibition has been reported in the auditory cortex of hearing-lesioned rat (Yang et al., 2011). In this model, GAD67 was reduced in the affected region, resulting in down-regulation of both phasic and tonic inhibition. In addition, hearing-lesioned animals displayed tinnitus with a pitch in the hearing loss range, which was eliminated by the GABA transaminase inhibitor, vigabatrin, or by reducing the uptake of GABA by the GAT1 inhibitor, NO711.

## COGNITIVE DYSFUNCTION AND FUNCTIONAL RECOVERY AFTER STROKE

Because cognition involves a group of mental processes such as memory, attention, understanding language, and learning, numerous brain regions are thus responsible for cognitive dysfunctions. The hippocampus plays a critical role in learning and memory, and recent evidence suggests that the α5-GABA_A_ receptor is abundantly expressed in this brain region, and thus may be a therapeutic target for cognitive dysfunction (Sperk et al., 1997).

Down syndrome is caused by the trisomy for chromosome 21, and is the most frequent neurodevelopmental disorder of chromosomal origin. Almost all affected individuals exhibit cognitive impairment, with specific deficits in learning and memory mediated by hippocampal dysfunctions (Carlesimo et al., 1997). This phenotype is well replicated in the Ts65Dn mouse model of Down syndrome. The finding that low-dose picrotoxin restored hippocampal long-term potentiation in this model (Kleschevnikov et al., 2004) subsequently led to the hypothesis of “excessive inhibition,” and was strongly recognized to play a pathological role in this syndrome. In support of this hypothesis, Ts65Dn mice were found to exhibit increased immunoreactivity of GAD65/67 and vesicular GABA transporter (vGAT; Belichenko et al., 2009; Perez-Cremades et al., 2010; Martinez-Cue et al., 2013). Numerous studies indicate that α5-GABA_A_ receptor-mediated inhibition (mainly in the tonic form) reduces learning and memory. Mice deficient in the α5 subunit show better performance of cognitive functions compared with wild-type (Collinson et al., 2002; Crestani et al., 2002; Yee et al., 2004). Similarly, rodents systemically administrated with various α5 subunit-selective negative allosteric modulators displayed a facilitation in learning and memory tasks, associated with increased long-term potentiation (Navarro et al., 2002; Atack et al., 2006,^2009^; Dawson et al., 2006; Ballard et al., 2009). Therefore, specific antagonism of the α5 subunit may be a potential therapeutic strategy against cognitive impairment in Down syndrome. Indeed, recent studies have revealed that cognitive function is ameliorated with the α5 subunit-selective negative allosteric modulators, α5IA, or RO4938581, in Ts65Dn mice without any side effects (Braudeau et al., 2011; Martinez-Cue et al., 2013). RO4938581 is currently in clinical trials for adults with Down syndrome (the study is sponsored by Roche, and its outcomes have been made publicly available). The α5-selective negative allosteric modulators have also been shown as effective enhancers for learning and memory in other cognitive deficits induced by acute inflammation (Wang et al., 2012) or general anesthesia (Saab et al., 2010; Zurek et al., 2012) in mice, and by alcoholism in humans (Nutt et al., 2007; Atack, 2010).

In the aged brain, hippocampal tonic inhibition can regulate cognitive functions. Recent animal studies and human brain imaging of elderly individuals have indicated a strong positive correlation between hippocampal hyperactivity and cognitive impairment (Wilson et al., 2005; Ewers et al., 2011). In support of the “excessive excitation” hypothesis, the positive (and not negative) allosteric modulator for the α5 subunit improves memory tasks in aged rats with cognitive deficits (Wilson et al., 2005; Koh et al., 2013). This hypothesis may be applied to autism spectrum disorders because antiepileptic drugs sometimes improve cognitive functions in these affected individuals (Di Martino and Tuchman, 2001). α5 subunit selective allosteric modulators currently in clinical trials have shown promise as a potential therapeutic strategy for cognitive deficits in various CNS diseases.

Several reports have demonstrated that the inhibitory balance between phasic and tonic inhibition shifts to tonic after cortical lesion (Clarkson et al., 2010; Imbrosci et al., 2012). This shift may provide protection against cell death in the acute stage, and also prevent homeostatic plasticity during the recovery period. In a cortical stroke model, excessive tonic inhibition has been proposed as a novel pharmacological target for promoting recovery after stroke (Clarkson et al., 2010). Therefore, negative allosteric modulators for the α5 subunit may be effective against learning and memory deficits as well as for dysfunctions after brain injury.

## PSYCHIATRIC DISORDERS

Schizophrenia is generally recognized to develop from the disruption of various kinds of neurotransmission. Dysfunction in GABAergic transmission has drawn significant attention as a mechanism underlying cognitive deficits in schizophrenia (Guidotti et al., 2005; Lewis et al., 2005). Postmortem analyses and brain imaging of living schizophrenic subjects have revealed a deficiency in the synthesis of GABA resulting from reduced transcription of GAD67 (Akbarian et al., 1995b; Volk et al., 2000; Hashimoto et al., 2003; Yoon et al., 2010). Results of studies investigating the expression of GABA_A_ receptor subunits in postmortem tissue have been inconsistent. The expression of α1, α2, and α5 subunits have been reported to be increased (Impagnatiello et al., 1998; Ishikawa et al., 2004; Lewis et al., 2004), decreased (Hashimoto et al., 2003), or unchanged (Akbarian et al., 1995a). Despite the limited number of reports investigating the expression of the δ subunit, several reports have shown decreased expression of this subunit in the prefrontal cortex (Hashimoto et al., 2003; Maldonado-Aviles et al., 2009). A considerable limitation of the postmortem tissue analyses in these studies is the chronic consumption of antipsychotics and/or benzodiazepines in the subjects. Furthermore, symptoms of schizophrenia dynamically change with the clinical stage. A recent study involving positron emission tomography (PET) using the highly selective α5 subunit ligand, [11^C^]Ro15-4513, revealed an inverse correlation between binding potential and severity of the negative symptoms in medication-free subjects (Asai et al., 2008). Therefore, this result suggests that decreased expression of the α5 subunit may be involved in the pathophysiology of schizophrenia during the negative stage. Supporting this hypothesis, use of the positive allosteric modulator for the α5 subunit has been shown to improve the hyperactivity and cognitive dysfunctions in rat models of schizophrenia (Damgaard et al., 2011; Gill et al., 2011). However, Redrobe et al. (2012) have shown that a negative allosteric modulator of the α5 subunit also attenuates cognitive deficits, using a different mouse model of schizophrenia. Interestingly, the authors also showed that hyperactivity was ameliorated by both positive and negative modulators for the α5 subunit in these mice. Excitatory and inhibitory biphasic changes induced by tonic conductance have been recently shown in single neurons with depolarizing reversal potential for GABA currents (Song et al., 2011). Therefore, an increase or decrease of tonic inhibition may result in net inhibitory effects in relevant neuronal circuits in this model. Further studies are required to validate the usefulness of α5 subunit selective allosteric modulators for the treatment of schizophrenia.

Neurosteroids, metabolites of progesterone, other sex hormones, and several stress-induced steroids are potent modulators of GABA_A_ receptors (Lambert et al., 2003). In particular, the extrasynaptic δ-GABA_A_ receptor shows higher sensitivity for neurosteroids compared with synaptic GABA_A_ receptors (Davies et al., 1997; Stell et al., 2003; see review Brickley and Mody, 2012). Therefore, dysregulation of tonic inhibition may play a pivotal role in psychiatric disorders, such as anxiety and mood disorders, which are often associated with sex hormone alterations or stress. For example, expression of the δ subunit has been shown to decrease during pregnancy due to adaptive mechanisms against large elevation of progesterone metabolites (Maguire and Mody, 2008). The rapid decrease of neurosteroids after birth can thus induce the down-regulation of tonic inhibition, resulting in postpartum depression (Maguire and Mody, 2008).

In a rat model of premenstrual dystrophic disorders, withdrawal of progesterone leads to anxiety and increased seizure susceptibility, associated with up-regulation of the α4 subunit (Smith et al., 1998). In contrast, increased levels of neurosteroids during physiological ovarian cycles leads to reduction of anxiety and seizure susceptibility caused by up-regulation of the δ subunit (Maguire et al., 2005). This difference may thus suggest that neurosteroids can alter the effects of tonic GABA conductance by dose and/or exposure time-dependent mechanisms, thereby regulating the expression of receptor subunits. In accordance with this speculation, evaluating anxiety at puberty revealed that neurosteroids negatively modulated tonic inhibition by decreasing outward currents, via α4δ-GABA_A_ receptors (Shen et al., 2007). This regulation was shown to contribute to psychiatric disorders during puberty, such as mood swings, anxiety, and anorexia (Aoki et al., 2012).

Excessive stress leads to physiological and behavioral responses resulting in numerous psychiatric disorders. The hypothalamic-pituitary-adrenal (HPA) axis plays an important role for mediating physiological responses by promoting stress-induced steroid synthesis (see review Kudielka and Kirschbaum, 2005). A recent study has demonstrated that stress-derived neurosteroid acts on δ-GABA_A_ receptors of corticotrophin releasing hormone (CRH) neurons, biphasically modulating their excitability depending on the presence or absence of stress (Hewitt et al., 2009). The study showed that stress decreased the activity of the outward-directed K^+^, Cl^-^ co-transporter, potassium-chloride transporter 2 (KCC2), in CRH neurons, switching tonic GABA conductance from inhibitory to excitatory and resulting in increased activity of the HPA axis and elevated levels of corticosterone (Hewitt et al., 2009). Because blockage of corticosterone synthesis prevents stress-induced anxiety, this positive feedback loop may be one of the mechanisms underlying stress-induced psychiatric disorders (Sarkar et al., 2011). Overall, emerging evidence has uncovered a bidirectional modification of tonic inhibition by neurosteroids, and thus may explain why increases in neurosteroids can lead to various types of psychiatric disorders, including depression and anxiety.

## AUTISM SPECTRUM DISORDERS AND RELATED SYNDROMES

“Autism spectrum” encompasses a wide range of disorders, which can be divided into two groups with respect to their etiology: (1) recognizably distinct syndromes caused by mutations in specific genes or chromosomal loci, and (2) more common, genetically heterogeneous subjects, referred to as idiopathic autism (Coghlan et al., 2012). The number of subjects in the first group is much lower than idiopathic autisms (Coghlan et al., 2012), however their pathophysiology can be rigorously analyzed by using a genetic mouse model.

**FIGURE 2:**
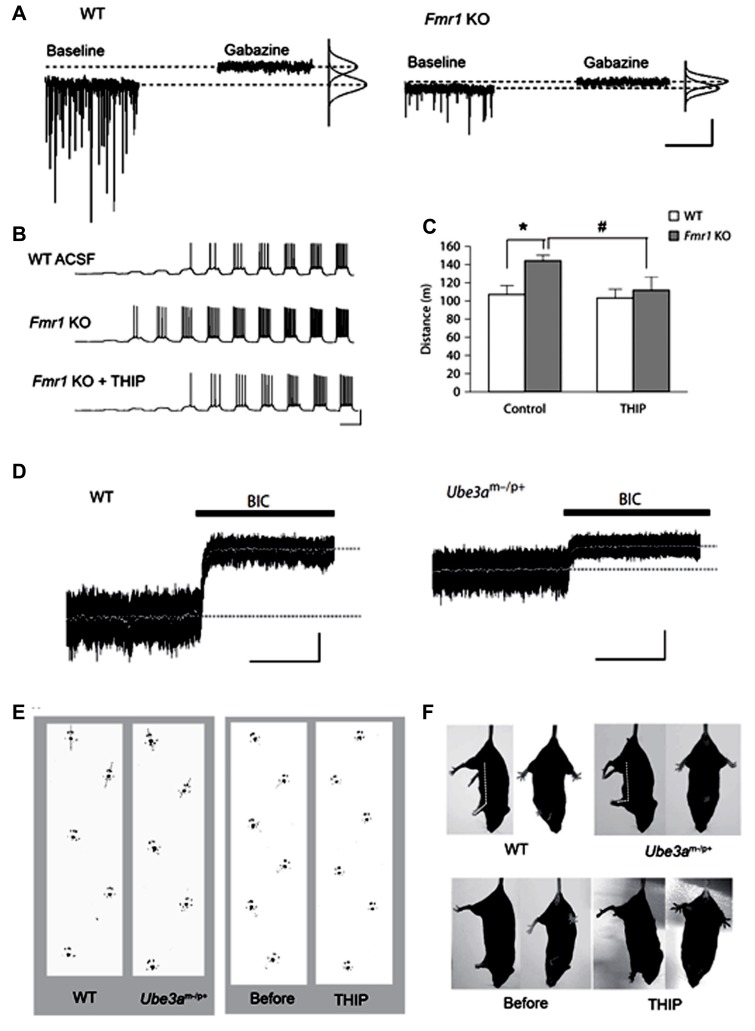
**Decreased tonic inhibition in mouse models of the neurodevelopmental disorders, fragile X syndrome (FXS) and Angelman syndrome**. **(A–C)** Analyses in fragile X mental retardation 1 (Fmr1) knock out (KO) mice (FXS model). **(A)** Voltage clamp traces from principal neurons of amygdala in wild type (WT) and Fmr1 KO mice, showing impaired phasic and tonic inhibition in KO mice. **(B)** Fmr1 KO mice have higher action potential (AP) firing rates for a given depolarizing current step and a lower threshold for AP generation (middle panel), which is reverted to WT levels with THIP (lower panel). **(C)** In the open field test, THIP prevents hyper locomotor activity in Fmr1 KO mice. *p < 0.05, WT control vs. Fmr KO control; #*p* < 0.05, Fmr1 KO control vs. Fmr KO THIP. **(D,E)** Analyses in ubiquitin-protein ligase E3A (Ube3a) KO mice (Angelman syndrome model). **(D)** Decreased cerebellar tonic inhibition in Ube3a KO mice [black bars: 20 μM bicuculline (BIC)]. **(E)** Ataxic gait rescued by administration of THIP (right panel) in Ube3a KO mice. **(F)** THIP (lower panels) improves clasping reflex (via the tail-suspension test) in Ube3a KO mice. **(A–C)** Adapted from Olmos-Serrano et al. (2010),^2011^; **(D–F)** adapted from Egawa et al. (2012).

Fragile X syndrome (FXS) is a neurodevelopmental disorder caused by malfunction of the fragile X mental retardation 1 (FMR1) gene on the X chromosome, which encodes the FMR protein (FMRP; Verkerk et al., 1991). Common symptoms include intellectual disability, a distinctive physical phenotype, and autism-like behavior often associated with epilepsy. FMRP is known to regulate the mRNA transcription of various synaptic plasticity-associated proteins in concert with activation of metabotropic glutamate receptors (mGluR; Bassell and Warren, 2008). mRNA of some GABA_A_ receptor subunits, particularly the δ subunit, are targeted substrates of FMRP (Miyashiro et al., 2003; Dictenberg et al., 2008). Dendritic localization of δ subunit mRNA has been shown to be regulated by mGluR activation under the presence of FMRP (Dictenberg et al., 2008), suggesting that tonic inhibition plays an important role for the homeostatic regulation of excitation-inhibition during development. Indeed, electrophysiological analysis in FMR1 KO mice revealed that tonic, but not phasic, inhibition in subicular pyramidal neurons is significantly decreased, with decreased expression of δ and α5 subunits in this region (Curia et al., 2009). Decreased expression of the δ subunit protein has also been shown in the cortex and hippocampus of this mouse model (El Idrissi et al., 2005). Olmos-Serrano et al. (2010) demonstrated that FMR1 KO mice exhibit remarkably reduced tonic and phasic inhibitory currents in the amygdala principal neurons, with reduced GAD65 and GAD67 expression. This group also showed that THIP rescued neuronal hyperexcitability, *in vitro*, and ameliorated hyperexcitability, *in vivo* (Olmos-Serrano et al., 2010,^2011^; **Figures 2A–C**), indicating that tonic inhibition is a potent therapeutic target for FXS. In contrast, increased GAD65 and GAD67 expression was also observed in the cortex and hippocampus of FMR1 KO mice (El Idrissi et al., 2005; Adusei et al., 2010), thus indicating regional differentiation of deregulation mechanisms of inhibition.

Similar presynaptic dysfunction in GABAergic neurotransmission has been proposed as a mechanism underlying the autism spectrum-related disorder, Rett syndrome. Mice lacking the causal gene, methyl CpG binding protein 2, from GABAergic neurons recapitulate phenotypes as those seen in Rett syndrome, with decreased GABA levels, reduced expression of GAD65/67, and miniature inhibitory postsynaptic current amplitudes in cortical pyramidal neurons (Chao et al., 2010). Therefore, reduction of tonic inhibition may also contribute to the pathophysiology of Rett syndrome.

Findings from our recent study have shown decreased tonic inhibition as a mechanism underlying cerebellar ataxia in the autism spectrum-related disorder, Angelman syndrome (Egawa et al., 2012). This syndrome is one of the ubiquitin-proteasome pathway-associated diseases with the causal gene, ubiquitin-protein ligase E3A (*UBE3A*), encoding the Ube3a protein (Kishino et al., 1997). We found that Ube3a binds to GAT1 and directly controls its degradation in the cerebellum (Egawa et al., 2012). Furthermore, a surplus of GAT1 was found to decrease tonic currents of CGCs in Ube3a KO mice. Because THIP can improve cerebellar dysfunctions in this mouse model under *in vitro* and *in vivo* conditions (**Figures 2D–F**), the reduction of tonic inhibition could thus underlie cerebellar ataxia in Angelman syndrome. Expression of GAT1 and Ube3a is increased during development (Takayama and Inoue, 2005; Sato and Stryker, 2010). Therefore, our findings suggest that Ube3a-dependent degradation of GAT1 plays an important role for neuronal plasticity of inhibitory systems by regulating tonic inhibition. Disruption of CaMK II-mediated regulation of membrane trafficking of extrasynaptic GABA_A_ receptors may also result in decreased tonic inhibition, because deactivation of CaMK II is known to play a role in the pathophysiology of this syndrome (Weeber et al., 2003). Ube3a is widely expressed in the CNS (Jiang et al., 1998), thus decreased tonic inhibition may also be responsible for other symptoms, such as autistic behavior, mental retardation, and seizures. Notably, Angelman syndrome is predominantly caused by the large deletion of the relevant chromosome, 15q11-13, containing genes that encode the GABA_A_ receptor α5, β3, and γ3 subunits. Therefore, these individuals show a greater severity in phenotypes compared with individuals without the deletion (Lossie et al., 2001; Egawa et al., 2008). The relative expression of β3 and α5 was shown to be reduced in postmortem brain tissue from patients lacking these genes (Roden et al., 2010). Therefore, tonic inhibition may be lower in typical Angelman syndrome individuals with the large deletion of 15q11-13, which could result in the phenotypic difference in patients with or without the deletion.

Accumulating evidence has indicated that the chromosome 15q11-13 abnormalities are highly correlated with the prevalence of idiopathic autisms. Genetic multi-linkage analysis has identified GABA_A_ receptor α5 and β3 subunit genes on 15q11-13 that contribute to autism susceptibility (McCauley et al., 2004). Furthermore, this region is one of the most common loci where copy number variants have been observed in idiopathic autisms. The prevalence of 15q11–13 duplications in idiopathic autisms has been estimated to be at 3% (Coghlan et al., 2012). Although an increased copy number of 15q11–q13 has been predicted to elevate the gene expression of GABA_A_ receptor subunits, recent *in vitro* studies using human neuronal cell lines have shown contradictory results, found to be due to impaired homologous pairing (Meguro-Horike et al., 2011). Therefore, expression of GABA_A_ receptor α5 and β3 subunits is likely to be reduced in individuals with duplicated 15q11-13. Furthermore, attenuated expression of α5 and β3 subunits may be a common pathophysiological feature in idiopathic autisms without the 15q11-13 mutation. In support of this hypothesis, postmortem brain tissue analyses have reported that the expression of α5 and β3 subunits are significantly decreased (in four out of eight cases) in idiopathic autism spectrum disorders (Hogart et al., 2007). Further support was shown in a PET study in which binding of [11^C^]Ro15-4513 was significantly lower throughout the brain, particularly in the amygdala, in adult idiopathic autism patients who did not take any neuro-modulative medications (Momosaki et al., 2010). However, compensatory regulation that may reduce the expression of the α5 receptor in idiopathic autism subjects cannot be excluded. Nevertheless, findings from mouse models of specific gene-associated autism spectrum disorders and brain imaging of human subjects, suggest that reduced tonic inhibition may be one of the common mechanisms underlying neuronal dysfunctions in autism spectrum disorders.

## NON-SYNAPTICALLY RELEASED GABA INDUCES TONIC DEPOLARIZATION DURING CORTICOGENESIS

**FIGURE 3:**
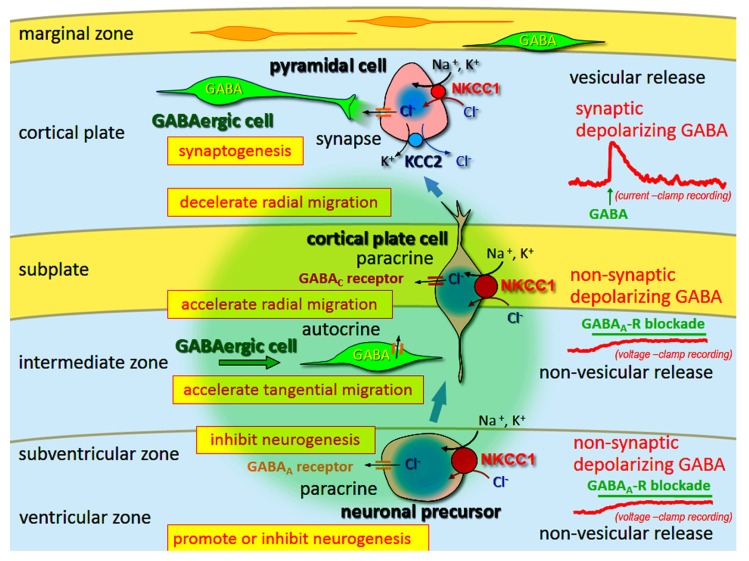
**Multi-modal actions of tonic modulation of the GABA_A_** receptor during corticogenesis. In the ventricular zone (VZ) and subventricular zone (SVZ), ambient GABA affects neurogenesis. Post-mitotic neurons migrating to the cortical plate are tonically depolarized by non-synaptic and non-vesicular ambient GABA, released from tangentially migrating GABA neurons prior to forming synapses, and changing migratory pace. GABA also acts in an autocrine manner to accelerate tangential migration. Vesicular release of GABA, which remains depolarizing, may contribute to synapse formation. Following the establishment of GABAergic synapses (and prior to hyperpolarization), GABA acts as an excitatory neurotransmitter due to high intracellular Cl^-^ concentration levels, predominantly resulting from Na–K–Cl cotransporter 1 (NCC1) followed by K–Cl cotransporter 2 (KCC2). Green circle: ambient GABA. Adapted from Fukuda and Wang (2013).

Gamma-aminobutyric acid induces depolarization and hyperpolarization in the immature and adult brain, respectively. The GABA_A_ receptor is a Cl^-^ channel (also permeable for HCO_3_^-^), and thus the developmental switch of GABA-mediated depolarization to hyperpolarization is induced by changes in the transmembranous Cl^-^ gradient, from its efflux to influx, respectively. This change is regulated by cation-Cl^-^ cotransporters [Na–K–Cl cotransporter 1 (NKCC1), Cl^-^ uptake; and KCC2, Cl^-^ extrusion]. Furthermore, in immature neurons the release mechanism of GABA is non-vesicular and non-synaptic, and therefore GABA-mediated activity is generally tonic. These tonic depolarizing (and excitatory on occasion) GABA actions are necessary for neurogenesis, differentiation, migration, and synaptogenesis (**Figure 3**).

During development, GABA_A_ receptor subunits exhibit a completely different expression pattern (Laurie et al., 1992; Fritschy et al., 1994) compared with that in the adult. At the prenatal stage and during early postnatal days, cortical neurons predominantly express α2, α3, α4, α5, β2, β3, γ1, and γ2 subunits (Laurie et al., 1992). Gene transcripts of α2, α3, and α5 subunits have been detected in the developing CNS (Laurie et al., 1992). At rat embryonic day 17 and 20, α4, β1, and γ1 subunit transcripts have been found to be abundant in the inner half of the germinal matrix corresponding to the ventricular zone (VZ), [although undetectable in the intermediate zone (IZ)], thus implying that proliferating cells may express these subunits (Ma and Barker, 1995).

GABA_A_ receptor-mediated signaling affects neurogenesis during development. A pioneering study by LoTurco et al. (1995) provided evidence that GABA can affect the proliferation of progenitor cells. They showed that depolarizing GABA actions led to a decrease in both DNA synthesis and the number of bromodeoxyuridine (BrdU)-labeled cells in the rat embryonic neocortex. However, later studies have revealed that GABA also has varying effects on neurogenesis in different cell-types and/or brain regions. Using organotypic slices of mouse embryonic neocortex, Haydar et al. (2000) revealed that differential regulation of cell production by depolarizing GABA activity occurs in cortical progenitor cells located in different regions [i.e., promotion and inhibition of cell division in the VZ and subventricular zone (SVZ), respectively]. These opposing effects may be attributed to the difference in the subunits of GABA_A_ receptors or in the regional concentration of ambient GABA (Morishima et al., 2010).

In the neocortex, different classes of neurons, pyramidal cortical neurons, and GABAergic interneurons, originate from different sources and have distinct migration routes. Glutamatergic pyramidal neurons radially migrate from the dorsal telencephalic VZ where they are generated via radial glial scaffolding toward the cortical plate. In contrast, GABAergic interneurons arising from the medial ganglionic eminence (MGE) tangentially migrate into the cerebral wall (for review, see Marïn and Rubenstein, 2001). GABA_A_ receptor signaling is well known to have distinct effects on radially or tangentially migrating neurons. Behar et al. (1996) demonstrated that the GABAergic modulation of neuronal migration was strongly concentration-dependent, in which femtomolar concentrations of GABA stimulated the migration along a chemical gradient (i.e., chemotaxis) while micromolar concentrations increased random migratory movement (i.e., chemokinesis). Furthermore, both processes were shown to be mediated by an increase in intracellular calcium, suggesting that GABA mediates chemotactic as well as chemokinetic migratory responses in embryonic neocortical neurons. The effect of GABA on migration is dependent on its location and receptor. Activation of neuroblastic GABA_B_ or ρ-subunit-containing GABA_A_ receptors promotes GABA migration from the SVZ and IZ, and the activation of GABA_A_ receptors in cortical plate cells induces a stop signal (Behar et al., 2000; Heck et al., 2007; Denter et al., 2010). GABA also regulates the tangential migration of immature GABAergic cortical interneurons. After generation in the MGE, GABAergic interneurons migrate to the cortex via the corticostriatal junction (CSJ), avoiding the striatum (Cuzon et al., 2006). Although ambient GABA levels in the CSJ are similar to those in the MGE, the response of tonic GABA_A_ receptor to ambient GABA is likely to be enhanced in the CSJ region, suggesting that a dynamic expression of GABA_A_ receptor isoforms on MGE-derived cells promotes the cortical entry of tangentially migrating MGE-derived cells (Cuzon and Yeh, 2011). When GABAergic interneurons terminate their migration in the cortex their response to ambient GABA changes to a stop signal via up-regulation of KCC2 (Bortone and Polleux, 2009).

Tonic activation of GABA_A_ receptors precedes phasic activation in early development (for review, see Owens and Kriegstein, 2002), hence its possible requirement for synaptogenesis during this period (Demarque et al., 2002; Liu et al., 2006). Spontaneous depolarizations of GABA would be the first excitatory drive required for activity-dependent synaptogenesis (Nakanishi et al., 2007; Wang and Kriegstein, 2008; for review, see Ben-Ari et al., 2007).

## PERTURBATION OF TONIC DEPOLARIZATION MAY CAUSE BRAIN MAL-DEVELOPMENT

As mentioned previously in this review, tonic depolarizing responses to ambient GABA is locally controlled by uptake and/or release mechanisms allowing GABA_A_ receptor-mediated actions to control a variety of developmental events in a time- and region-specific fashion. Thus, a defect in GABA_A_ receptor-mediated multimodal functions during development can cause neuronal circuit dysfunctions hence neurological disorders. Therefore, use of GABA_A_ receptor activating drugs to enhance inhibition in the immature brain may result in unexpected deleterious effects. A growing number of studies in immature animal models have demonstrated degenerative effects of several anesthetics on neuronal structure and brain functions, such as social behaviors, fear conditioning, and spatial reference memory tasks (Jevtovic-Todorovic et al., 2003; Loepke et al., 2009; Satomoto et al., 2009). Several animal studies have demonstrated that sevoflurane (Satomoto et al., 2009) and isoflurane (Sall et al., 2009; Stratmann et al., 2009), which bind to GABA_A_ receptors, exhibit deleterious effects on neuronal survival and neurogenesis when exposed at early periods of life.

Neocortical malformations, such as polymicrogyria show the presence of cortical cells in heterotopic positions; often caused by a disruption in neuronal migration (Walsh, 1999; Francis et al., 2006). Therefore, perturbation of the numerous functions of GABA during development may underlie the etiology of cortical malformations. Focal freeze lesion in the cerebral cortex of newborn (P0) rats was shown to produce microgyrus, a focal cortical malformation with a small sulcus and a 3- or 4-layered microgyric cortex. This defect resembles human 4-layered polymicrogyria (Dvorák and Feit, 1977), a clinical condition that results from abnormal neuronal migration. Wang et al. (2013) have reported that temporally increased ambient GABA causes tonic activation of neuronal GABA_A_ receptors (possibly due to depolarization because NKCC1 is up-regulated and KCC2 is down-regulated), resulting in the modulation of intracellular Ca^2^^+^ oscillations. These oscillations could differentially affect the migratory status of GABAergic cells (“go”) and cortical plate cells (“stop”). Therefore, immature cortical plate neurons and GABAergic neurons migrate in distinct patterns to form the microgyrus and thus, abnormally induced tonic functions of GABA could be involved in migration disorders that ultimately result in cortical malformations.

## CONCLUSION

This review has focused on the recent progress in understanding the impact of dysregulated tonic inhibition in CNS diseases. Accumulating evidence highlights the pathophysiological significance of dysregulated tonic inhibition in a number of neurodevelopmental disorders, psychiatric disorders, and cognitive dysfunctions (see **Figure 1**). In addition to pharmacological progress, animal model studies are presently opening the door for practical medicine. However, an overview of these studies also presents problems for clinical application. For example, the same phenotype may result from a decrease or increase of tonic inhibition. Therefore, the net shift from excitation to inhibition may not always occur with a modulation of tonic inhibition. This paradox is possibly due to the complexity of network regulation by tonic conductance. For example, simultaneous receptor activation in either inhibitory or excitatory neurons, or multiple mechanisms can be responsible for changing membrane properties, such as shunting inhibition and a shift in intracellular Cl^-^ concentration. Although temporal characteristics of tonic inhibition are weak, maintaining network functions can possibly regulate its strength, thus suggesting a narrow therapeutic range for such modulators. A detailed assessment of these mechanisms would therefore be needed to develop a therapeutic strategy.

This review also discussed tonic depolarization induced by ambient GABA during early development, where the release of GABA is possibly non-vesicular and occurring before synaptic organization (see **Figure 3**). Thus far, very few studies have addressed this series of events as a potential risk factor for CNS diseases. Therefore, research focused on the pathological effects of multimodal functions of GABA during neurodevelopment may shed light on understanding the pathology of neurodevelopmental disorders in which GABA is known to play a role, for example in schizophrenia and autism spectrum disorders.

## Conflict of Interest Statement

The authors declare that the research was conducted in the absence of any commercial or financial relationships that could be construed as a potential conflict of interest.
